# Editorial: The Molecular Mechanisms of Antibiotic Resistance in Aquatic Pathogens

**DOI:** 10.3389/fcimb.2020.586460

**Published:** 2020-09-24

**Authors:** Xiangmin Lin, Patrícia Poeta, Bo Peng

**Affiliations:** ^1^School of Life Sciences, Fujian Agriculture and Forestry University, Fuzhou, China; ^2^Microbiology and Antibiotic Resistance Team (MicroART), Department of Veterinary Sciences, University of Trás-os-Montes and Alto Douro (UTAD), Vila Real, Portugal; ^3^School of Life Sciences, Sun Yat-sen University, Guangzhou, China

**Keywords:** aquatic pathogens, antibiotic resistance, mechanisms, multidrug resistance, prevention strategies

Bacterial antibiotic resistance has become a formidable problem in the treatment of infectious diseases worldwide. The molecular mechanisms of antibiotic resistance remain elusive, especially for aquatic pathogens. Antibiotic resistance and virulence determinants in environmental/aquatic bacterial pathogens are the subject of five research articles published in this Research Topic. Mishra et al. have investigated the influence of outer membrane proteins (OMPs) on the antibiotic resistance profile and virulence factors of *Enterobacter* isolated from aquatic environments and clinical settings. The environmental *Enterobacter* isolates lack OmpC, are unable to invade host cells, and induce low amounts of reactive oxygen species (ROS) production by neutrophils. In contrast, a clinical *Enterobacter* isolate described possesses OmpF, has better invasive and adhesive capabilities, and stimulates a higher amount of ROS production. Furthermore, some clinical *Enterobacter* isolates which have the *ompA* gene also have the strong capacity to form biofilms. Taking a different angle, Abdelhamed et al. and Opazo-Capurro et al. have sequenced the whole genomes of the *Edwardsiella piscicida* MS 18-99 strain possessing a 117.4 kb conjugative plasmid pEPMS-1899, and *Acinetobacter radioresistens* A154 harboring several plasmids, respectively. The genome analysis revealed that several antimicrobial resistance genes/elements confer resistance to various antibiotics, such as *tetA&R, strA&B, sul2*, and *floR* in MS 18-99, *arsA* and *arsD* on pEPMS-1899, and *tetB, strA, strB, aph(3*′*)-Vla, aac(3)-IIa, blaSCO-1, blaTEM-1B*, and *blaPER-2* in *A. radioresistens*. A study by Montso et al. describes the presence of genes such as *shiga toxins* (*stx*) and *bundle-forming pili* (*bfpA*) that are related to virulence determinants and antibiotic resistance in atypical enteropathogenic *Escherichia coli* (aEPEC) O177, the first reported occurrence of the aEPEC O177 strain in South African cattle. Tekedar et al. have characterized genome sequences of *Aeromonas veronii* MS 17-88 strains isolated from channel catfish to uncover the mechanism of multidrug resistance. This strain has several antimicrobial resistance genes/elements such as *tet(34), tet(35), tet(E), tetR, mcr-7.1*, and *floR*. These five studies have all contributed further evidence of the abundance and prevalence of antibiotic resistance genes in different bacterial genomes.

Proteins are the fundamental and vital material of life. Ribosome rescue is a mechanism to maintain protein synthesis when mistakes or stalling occur. Quality control of protein synthesis is part of this process, which requires transfer-messenger RNA (tmRNA) and small protein B (SmpB). In this Research Topic, there are three articles from Prof. Liu's group describing the involvement of tmRNA and SmpB in the antibiotic resistance and persistence development of *A. veronii*. The transcriptomic data presented reveal the downregulation of protein secretion systems, such as the type VI secretion system (T6SS) and type IV pilus assembly, in the SmpB deficient strain. This downregulation attenuated the production of virulence factors and the colonization ability of *A. veronii*. However, the alternative rescue factor A (AvrA) acts as a substitute for virulence compensation. The decreased level of *avrA* expression increases the expression level of iron-sulfur protein activator *iscR*, resulting in an increase in bacterial antioxidant ability through enhancement of the synthesis of the iron-sulfur protein and assembly of iron-sulfur clusters (Santos et al., 2014). SmpB deficiency showed attenuated virulence and enhanced tolerance to oxidative stress. In another study, the deletion of the *smpB* gene in *A. veronii* enhanced the expression of enzymes related to adenosine metabolism, which leads to increases in the products such as adenosine AMP, cAMP and deoxyadenosine. By comparison, the deletion of *ssrA* (tmRNA) upregulated the expression of guanosine metabolism and hypoxanthine synthesis, eventually increasing the levels of the metabolic product xanthine, which in turn downregulated the genes related to the efflux pump such as *acrA* and *acrB*. With the *ssrA* and *smpB* genes deleted, the minimal inhibitory concentration of trimethoprim against *A. veronii* was lower. Therefore, this study demonstrates the effect of SmpB and tmRNA on different branches of purine metabolism and tolerance to trimethoprim (Wang et al.). A study by Yu et al. showed that the deletion of *ssrA* (tmRNA) increased the capacity of *A. veronii* to form persister cells. Moreover, Δ*ssrA* significantly raised the intercellular levels of N-acetylglucosamine production, promoting NaCl osmotic stress tolerance. To confirm this, the authors exogenously added N-acetylglucosamine to the medium and observed resistance to osmotic pressure and higher persistence to cefotaxime in *A. veronii*. Overall, these three research articles suggest that the trans-translation system might be an effective target in the treatment of *A. veronii* and other multidrug resistant aquatic bacterial pathogens.

Enhancing the efficiency of traditional antibiotics is a vital strategy for combatting the antibiotic resistance in bacterial pathogens. A study by Sun et al. has revealed that indole and its derivative 2,5-methylindole potentiate the killing efficacy of tobramycin against persister cells of *Staphylococcus aureus*. Furthermore, combinations of indole and its derivatives with aminoglycoside antibiotics were effective in inhibiting the growth of many Gram-positive bacterial pathogens such as *Streptococcus pyogenes* and *Enterococcus faecalis*. Finally, Zhou et al. have studied the role of the type III secretion system (T3SS) on the pathogenicity of *Vibrio alginolyticus*. Swarming motility is inhibited in the T3SS mutant strain *tyeA* and its virulence toward zebrafish is also reduced. Further, the *tyeA* mutant strain is extremely susceptible to cefoperazone, minocycline, amikacin, and gentamicin. The effects of a live attenuated vaccine made from the mutant strain on the survival of zebrafish was tested. The data presented show that the survival of zebrafish infected with mutant was enhanced compared to the control infected with the wild-type strain. Both of these approaches may pave the way for the development of an effective treatment strategy to combat antibiotic resistance in bacterial pathogens.

## Author Contributions

XL and PP wrote the manuscript. BP revised and approved the final version of the manuscript. All authors conceived the outline of the manuscript.

## Conflict of Interest

The authors declare that the research was conducted in the absence of any commercial or financial relationships that could be construed as a potential conflict of interest.
